# Mom sweet, baby sad

**DOI:** 10.1093/lifemeta/loaf030

**Published:** 2025-07-31

**Authors:** Dongliang Lu, Xun Huang

**Affiliations:** State Key Laboratory of Metabolic Dysregulation & Prevention and Treatment of Esophageal Cancer, Tianjian Laboratory of Advanced Biomedical Sciences, Academy of Medical Sciences, Zhengzhou University, Zhengzhou, Henan 450052, China; State Key Laboratory of Metabolic Dysregulation & Prevention and Treatment of Esophageal Cancer, Tianjian Laboratory of Advanced Biomedical Sciences, Academy of Medical Sciences, Zhengzhou University, Zhengzhou, Henan 450052, China; Institute of Genetics and Developmental Biology, Chinese Academy of Sciences, Beijing 100101, China; State Key Laboratory of Metabolism and Regulation in Complex Organisms, Taikang Center for Life and Medical Sciences, School of Basic Medical Sciences, Wuhan University, Wuhan, Hubei 430071, China

**The widely consumed high-fructose corn syrup (HFCS) has been linked to neurological disorders. In a recent article published in *Nature*, Wang *et al*. extend the harms of excessive fructose intake on neurodevelopment to the prenatal and lactation stages. They reveal that fructose uptake via GLUT5 reprograms microglial metabolism towards a low-ATP production state, suppressing microglial phagocytosis and impairing neurodevelopment.**

Dietary nutrients have been found to exert widespread effects on brain function, including appetite regulation, neural function, and brain development [1, 2]. Among these nutrients, fructose has gained increasing attention due to its harmful effects on the brain, particularly in light of the rising consumption of high-fructose corn syrup (HFCS) in recent decades. High fructose intake has been shown to induce mitochondrial dysfunction, oxidative stress, and neuroinflammation in the adult brain [3], effects that are thought to be mediated, in part, by the accumulation of fructose-derived advanced glycation end-products (AGEs) in neurons [4]. In addition to its neurological effects, excessive fructose consumption has been linked to metabolic disorders such as obesity, insulin resistance, and cardiovascular disease [5].

The health risks of fructose are closely related to its unique metabolism in cells. Unlike glucose, fructose bypasses the rate-limiting phosphofructokinase (PFK)-dependent step and is directly phosphorylated by fructokinase to form fructose-1-phosphate (F1P). F1P is subsequently broken down into glycolytic intermediates such as dihydroxyacetone phosphate (DHAP) and glyceraldehyde, which can enter gluconeogenesis and adipogenesis pathways, or be further catabolized into lactate, acetyl-CoA, alanine, and oxaloacetate [6]. Due to the absence of feedback inhibition in this metabolic pathway, high fructose exposure can lead to an overproduction of downstream metabolites and trigger metabolic reprogramming. This dysregulation often disrupts cellular homeostasis and impairs normal cellular function.

Multiple recent studies have also reported that high fructose consumption damages the hippocampus in young animals and children [7, 8], suggesting that fructose may hinder neurodevelopment. Early life, especially the prenatal and lactation periods, represents a critical window for brain development. However, how fructose affects neurodevelopment during this period remains poorly understood. A recent study by Wang *et al*. revealed that high fructose intake during early life directly impairs microglial phagocytic function, leading to cognitive deficits and anxiety-like behavior [9].

Given that microglia are the only brain cell type expressing the fructose transporter GLUT5 (glucose transporter type 5, encoded by *Slc2a5*), and that microglial efferocytosis is essential for clearing dying neurons during brain development [10], the authors first investigated how fructose affects microglia in neonatal mice. In neonatal mice exposed to high fructose, either via intragastric administration or through maternal consumption during pregnancy and lactation, the study found reduced microglia numbers and diminished phagocytic activity. Notably, even fructose exposure limited to gestation or lactation alone was sufficient to impair microglial phagocytosis, indicating that maternal fructose consumption may disrupt the neonatal brain.

Then the indispensability of GLUT5 in these effects was determined. In Slc2a5 (GLUT5)-deficient mice and primary microglia, GLUT5 knockout completely reversed the fructose-induced reduction in microglia number, altered morphology, and impaired phagocytic capacity, demonstrating that fructose directly and negatively affects microglia through GLUT5-mediated uptake. By tracing [2-¹³C]-fructose metabolism, the study further confirmed increased fructose catabolism in microglia, with elevated levels of lactic acid and glutamic acid. By analyzing the fructose uptake and catabolism in different conditions, the authors observed that the levels of these fructolysis products were inversely correlated with microglial phagocytic activity, suggesting that metabolic changes underlie the functional impairment.

Interestingly, fructose exposure also increased the levels of fructose-6-phosphate, a metabolite not directly involved in fructose catabolism, suggesting the activation of alternative pathways such as hexokinase (HK)-mediated glycolysis. HK2 is a known negative regulator of phagocytosis, and its activation and mitochondrial localization are associated with reduced ATP production and impaired phagocytosis in microglia [11, 12]. This study showed that high fructose exposure promotes HK2 mitochondrial localization and reduces ATP levels in microglia, contributing to phagocytic dysfunction.

Finally, the study assessed the long-term behavioral consequences of early-life fructose exposure. Wild-type mice exposed to high fructose during development exhibited significant cognitive deficits and increased anxiety-like behavior, whereas these effects were absent in both global and microglia-specific GLUT5-deficient mice.

**Figure 1:**
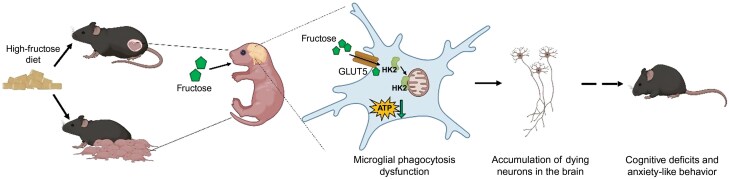
Excessive fructose consumption during pregnancy and lactation leads to high fructose exposure in neonates. In microglia, fructose uptake via GLUT5 increases HK2 localization to mitochondria and reduces ATP production, resulting in suppressed microglial phagocytosis. This leads to the accumulation of dying neurons in the brain, and ultimately causes cognitive deficits and increased anxiety-like behavior (created in BioRender.com).

In summary, this study provides compelling evidence that excessive fructose intake during early life impairs neurodevelopment by suppressing microglial clearance of dying neurons, a process dependent on the fructose transporter GLUT5. Mechanistically, fructose uptake reprograms microglial metabolism, enhances HK2 localization to mitochondria, reduces ATP production, and inhibits phagocytosis (Fig. 1). These findings highlight the neurodevelopmental risks of high fructose consumption during prenatal and neonatal periods and identify GLUT5 as a potential therapeutic target to mitigate the impact of dietary fructose on brain development.

Are there ways to mitigate the harmful effects of fructose intake beyond targeting GLUT5? The intestine and liver typically metabolize most dietary fructose [13], suggesting that harmful effects may be avoided if intake remains below a safe dosage. The threshold for harmful intake, particularly from HFCS, needs to be further investigated. Fructose-rich fruits do not show the same negative effects as HFCS, likely due to their high fiber and vitamin content. This raises the possibility that consuming fructose along with specific nutrient-enriched foods could reduce its toxic impact. Addressing these questions will provide valuable guidance for safer dietary fructose consumption in daily life.
